# Hospital Saturday Fund

**Published:** 1905-01-28

**Authors:** 


### HOSPITAL SATURDAY FUND.

At a meeting of the Hospital Saturday Fund, held at the central offices, Gray's Inn Road, last Saturday, Sir Savile Crossley, M.P., chairman of the fund, presiding, it was unanimously resolved on the recommendation of the Distribution Committee to award a sum of £21,861 13s. to 198 institutions. The total amount granted was apportioned as follows:—29 general hospitals, £7,736 19s.; 67 special hospitals, £6,693 6s.; 15 cottage hospitals, £287 13s.; 31 dispensaries, £953 4s.; 25 convalescent homes, £1,910 7s.; and 31 miscellaneous institutions £4,280 4s. The awards totalled £1,079 more than in the previous year. The total receipts for 1904 were £24,232.

